# Ronald D. Laings „radical trip“. Zum Verhältnis von Psychiatrie, Anti-Psychiatrie und Wissenschaft in den 1960er Jahren

**DOI:** 10.1007/s00048-022-00347-6

**Published:** 2022-10-17

**Authors:** Marina Lienhard

**Affiliations:** Universität Zürich, Zürich, Schweiz

**Keywords:** Antipsychiatrie, Gegenkultur, Schizophrenie, Familie, Entfremdung, Großbritannien, Antipsychiatry, Counterculture, Schizophrenia, Family, Alienation, Great Britain

## Abstract

Inspiriert von der amerikanischen Forschung zur Rolle des familiären Umfelds in der Entstehung der Schizophrenie begann der heute als Galionsfigur der britischen Antipsychiatrie bekannte schottische Psychiater Ronald D. Laing Ende der 1950er Jahre mit seinem Kollegen Aaron Esterson ein eigenes Forschungsprojekt. Dabei gelangte er zur Überzeugung, dass die als „schizophren“ Diagnostizierten weit vernünftiger seien als die von sich selbst entfremdeten bürgerlichen Familien. Angetrieben durch diese Perspektive drängte Laing stärker in die Öffentlichkeit und begann sich politisch zu engagieren. Dieser Beitrag analysiert Laings Entwicklung zum Anti-Psychiater und Star der Gegenkultur im Laufe der 1960er Jahre und fragt nach den Gründen und Möglichkeitsbedingungen für diesen Wandel vom Wissenschaftler zum „scientific political activist“.

## Abstract (en)

Inspired by American research on the role of the family environment in the development of schizophrenia, the Scottish psychiatrist Ronald D. Laing, now known as the figurehead of British antipsychiatry, began his own research project with his colleague Aaron Esterson in the late 1950s. In the process, he became convinced that those diagnosed as “schizophrenic” were far more rational than bourgeois families alienated from themselves. Driven by this perspective, Laing pushed harder into the public arena and began to become politically active. This article analyzes Laing’s development into an anti-psychiatrist and star of the counterculture in the course of the 1960s and asks about the reasons and conditions of possibility for this transformation from scientist to “scientific political activist”.

Im Sommer 1967 fand in Chalk Farm im Norden Londons während zweier Wochen ein schillerndes Ereignis statt. Unter dem Titel „Dialectics of Liberation“ trafen Expert*innen aus unterschiedlichsten Feldern aufeinander, um, wie es das Flugblatt beschrieb, über den gegenwärtigen Zustand der globalen Unfreiheit zu diskutieren.^1^ Black Power-Aktivist Stokely Carmichael traf hier auf den Philosophen Herbert Marcuse und den Beat-Poeten Allen Ginsberg. Die Anthropologen Gregory Bateson und Jules Henry waren ebenso zu Gast wie der Botaniker und Schriftsteller Francis Huxley und der Autor Paul Goodman. Organisiert wurde die Tagung von einer Gruppe in London ansässiger Psychiater und Psychoanalytiker^2^, die sich zu diesem Zweck als *Institute of Phenomenological Studies* zusammengeschlossen hatten. Darunter auch der schottische Psychiater Ronald D. Laing.

Laing hatte bis Anfang der 1960er Jahre eine relativ gewöhnliche Laufbahn verfolgt: Nach dem Medizinstudium in Glasgow und der Ausbildung zum Psychiater in verschiedenen Kliniken war er 1956 nach London gezogen, um sich zusätzlich als Psychoanalytiker ausbilden zu lassen und am *Tavistock Institute of Human Relations* zu forschen.^3^ Zum Zeitpunkt der „Dialectics of Liberation“-Tagung war er jedoch zu einer Galionsfigur der Londoner Gegenkultur und zu einem der wichtigsten Exponenten der sogenannten Antipsychiatrie geworden.^4^ Seine akademische Laufbahn hatte er weitgehend aufgegeben und stattdessen eine hierarchiefreie therapeutische Gemeinschaft in Kingsley Hall gegründet. Schizophrenie sei keine Krankheit, verkündete er nun, sondern ein sozialer Zustand, und der wahre Wahnsinn die von sich entfremdete bürgerliche Gesellschaft. Besonders für die gegenkulturelle Jugend wurde er so zur Projektionsfläche „alle[r] noch so sorgenvollen Sehnsüchte und eigenartigen Ideen [seiner] Zeit“, wie es 1976 in einem Radiobeitrag für den Hessischen Rundfunk hieß.^5^

Laing wandelte sich in den 1960er Jahren vom Psychiater zum Antipsychiater und damit, so möchte ich im Folgenden argumentieren, zum „scientific political activist“ entsprechend der in dieser Special Section vorgeschlagenen Definition: Zu einem aus der Wissenschaft kommenden Akteur also, der sein psychiatrisches Wissen und seine Forschung – jenseits der Rolle des beratenden Experten oder geisteswissenschaftlichen Intellektuellen – für gesellschaftliche Fragen außerhalb von Psychiatrie und akademischen Institutionen einsetzte. Über seine zunächst im wissenschaftlichen Mainstream angesiedelte Schizophrenieforschung gelangte er zu Überzeugungen, welche die Gesellschaft als Ganzes in Frage stellten und sich nicht mit einer konventionellen Karriere als Psychiater vereinbaren ließen. Er nutzte neue Kommunikationskanäle, um sich an eine breitere Öffentlichkeit zu wenden, und war an der Organisation verschiedener alternativer Foren beteiligt, die zu Hotspots der Londoner Gegenkultur wurden. Auch wenn er sich selbst nicht als „politisch“ im herkömmlichen Sinne verstand und sich damit von offen marxistischen Zeitgenossen abgrenzte, lassen sich seine Schriften, sein Auftreten und sein Handeln Mitte der Sechzigerjahre durchaus als gesellschaftliche Intervention verstehen, die eine Veränderung des Status quo herbeiführen sollte.^6^ Seine „politische Radikalisierung“ hing, wie ich zeigen werde, stark mit der Verwobenheit seiner Forschung mit aktuellen gesellschaftlichen Fragen zusammen und wurde zusätzlich durch mediale und institutionelle Bedingungen begünstigt. Wie sein amerikanischer Kollege Timothy Leary, der zeitgleich eine ähnliche Neuausrichtung durchlief, lässt sich Laings Persona in den 1960er und 1970er Jahren mit David Kaiser und Patrick McCray (2016: 5) als „Groovy Scientist“ beschreiben: eine selbst ernannte Guru-Figur mit einer hybriden Identität als Wissenschaftler und ein gegenkultureller Ikonoklast, dessen Wissenschaft geprägt war durch eine Mischung aus Verspieltheit und Ernsthaftigkeit und dem Gefühl, dass eine Revolution kurz bevorstehe (ebd.: 8).

Der Beitrag beleuchtet drei Phasen in Laings Werdegang und ist entsprechend chronologisch aufgebaut. Im Zentrum der ersten Phase steht Laings Auseinandersetzung mit der Schizophrenie ab Mitte der 1950er Jahre in seiner ersten Monographie *The Divided Self* und insbesondere seine Ausarbeitung und Durchführung eines Forschungsprojekts zusammen mit dem Psychiater Aaron Esterson, in dem sie 1958–1965 das Verhalten und die Kommunikation von Familien Schizophrener untersuchten. Der Prozess von der Konzeption des Projekts bis zur Veröffentlichung der Resultate im Band *Sanity, Madness and the Family *gibt einen Einblick in die Widersprüche und Kontextabhängigkeit von Laings Theoriebildung und zeigt, wo sich der spätere Antipsychiater von der bisherigen Forschung abzuheben begann. Die zweite Phase umfasst die Weiterentwicklung und Radikalisierung von Laings Theorien. Hier zeige ich auf, wie die Nutzung neuer Publikationsorgane Laing auch ein neues Publikum erschloss, was sich wiederum auf seine Theoriebildung auswirkte. Die dritte Phase wird gekennzeichnet durch Laings Neuausrichtung als „Groovy Scientist“ und politischen Akteur, die mit dem gesellschaftlichen Wandel ab Mitte der 1960er Jahre einherging. Am Beispiel Laings möchte ich also gewissermaßen die Genese eines „scientific political activist“ aufzeigen.

## Schizophrenie als Flucht aus der kleinbürgerlichen Familie

Um Laings Beschäftigung mit der Schizophrenie ab Mitte der 1950er Jahre und die Entwicklung seines Forschungsprojekts mit Esterson zu verorten, ist es wichtig, einen kurzen Blick in die Geschichte der ätiologischen Schizophrenieforschung zu werfen.

Nicht grundlos beschrieb der amerikanische Antipsychiater Thomas Szasz (1976) Schizophrenie spöttisch als heiliges Symbol der Psychiatrie. In der Frage nach dem Ursprung und dem Umgang mit der Schizophrenie kristallisierten sich im gesamten 20. Jahrhundert „grundsätzliche Fragen der psychiatrischen Epistemologie und Praxis“ (Maatz & Hoff 2017: 78) heraus. Darüber hinaus war Schizophrenie, sofern keine rein biologisch-organische Erklärung dafür angenommen wurde, bereits seit der Einführung der Diagnose durch den Schweizer Psychiater Eugen Bleuler 1908 mit gesellschaftlichen Fragen verknüpft. Bleuler verstand Schizophrenie unter anderem als „Assoziationsstörung“, das heißt als Störung des sozialen Anpassungsvermögens (Bernet 2013). Besonders in den USA hatte nach dem Zweiten Weltkrieg im Zuge eines allgemeinen Interesses für soziogene Erklärungen psychischer Krankheiten die Hypothese Konjunktur, dass die Entstehung der Schizophrenie durch bestimmte Arten zwischenmenschlicher Beziehungen begünstigt würde, oder, salopp ausgedrückt, dass Familien ihre Kinder in den Wahnsinn trieben.^7^ Dadurch wurde die Schizophrenie oder das schizophrene Kind als „Problemkind“ zum Austragungsort von Debatten zur „richtigen“ und „falschen“ Kindererziehung vor dem Hintergrund der gerade im Nachgang des Faschismus und während des Kalten Kriegs bedeutsamen Frage, wie ein mündiges, liberal-demokratisches „Subjekt“ erzogen werden könne.

Als Laing sich Mitte der 1950er Jahre theoretisch mit Schizophrenie zu befassen begann, handelte es sich also um ein sehr aktuelles Forschungsgebiet, das einerseits inmitten von größeren psychiatrischen „nature vs. nurture“-Debatten stand und andererseits mit gesellschaftlichen Fragen nach der Rolle der Familie und dem Zusammenhang von Psyche, Erziehung und Gesellschaftsformen verknüpft war. Diese Debatten fanden nicht etwa an den Rändern der Wissenschaft statt, sondern in deren Zentrum. Mit seinem Buch *The Divided Self*, das 1960 erschien, reihte sich Laing auf der „nurture“-Seite der Debatte ein. Als Anhänger Jean-Paul Sartres und Karl Jaspers verfolgte er das Ziel, Ansätze der Phänomenologie und des Existenzialismus mit Psychologie zu verknüpfen, um das Erleben Schizophrener so wiederzugeben, dass es nicht mehr unverständlich schien, sondern in Rückbindung an seinen Kontext erklärbar wurde. Vor diesem Hintergrund beschrieb Laing Schizophrenie als zwischenmenschliches Problem, das das Selbst zu zerstören drohte. Neu an seiner Perspektive war die Verwendung von philosophischen Konzepten und Begriffen, statt des psychiatrischen oder psychoanalytischen Jargons. Seine Perspektive auf die Schizophrenie wich jedoch nicht radikal von damaligen Lehrmeinungen ab. *The Divided Self* wurde fast durchwegs positiv rezensiert (Kotowicz 1997: 13). Außerdem sprechen die Verkaufszahlen dafür, dass das Buch für eine Fachpublikation durchaus breit rezipiert wurde (Beveridge 2011: 298).^8^ Anders als *The Myth of Mental Illness* von Thomas Szasz (1961), *Asylums* von Ervin Goffman (1961) und Michel Foucaults (1961) *Folie et déraison*, welche alle im Folgejahr erschienen, war *The Divided Self* nicht sonderlich psychiatriekritisch (Crossley 2006: 110) und ließ sich daher in den psychiatrischen Kanon einreihen (Kotowicz 1997: 13).

## Die Erforschung der „Schizogenese“

Während Laing in London am Manuskript von *The Divided Self *arbeitete, begann sich in den USA eine Forschungsrichtung durchzusetzen, die das Vorkommen von Schizophrenie in Familien untersuchte. Der Fokus lag dabei auf der Kommunikation und Interaktion der Familien. Ziel war es herauszufinden, welche Dynamiken und Muster „schizophrenogen“ wirkten. Mit kritischem Unterton beschrieb Autor und Aktivist Peter Sedgwick diesen Forschungstrend:

Hundreds of families have trooped into the laboratories of academic institutes and hospitals, there to have their entire verbal output tape-recorded over many sessions, their gestures and eye-movements filmed and their biographies unearthed in depth by interdisciplinary panels of doctors, psychologists, sociologists and technicians. (Sedgwick 1971: 16)

Den größten Effekt erzielte eine Publikation aus Palo Alto von 1956.^9^ Ein Team um den Anthropologen Gregory Bateson und den Psychiater und Psychotherapeuten Don Jackson veröffentlichte das Paper „Toward a Theory of Schizophrenia“, in welchem sie die sogenannte Double Bind-Theorie postulierten.^10^

Laing interessierte sich sehr für die Arbeit seiner amerikanischen Kollegen und begann Ende der 1950er Jahre zusammen mit seinem ehemaligen Kommilitonen an der Glasgow University, Aaron Esterson, ein eigenes Forschungsprojekt als britisches Pendant aufzuziehen.^11^ Methodisch wie inhaltlich hatte das Projekt starke Parallelen zu den amerikanischen Projekten: Durch Interviews der verschiedenen Familienmitglieder einzeln sowie in allen möglichen Konstellationen wollten sie hauptsächlich die Frage beantworten, welche Interaktionsmuster sich bei den Familien erkennen ließen und welche dieser Muster schizogen schienen. Die Nähe zur amerikanischen Forschung wurde dadurch verstärkt, dass Laing 1961 im Rahmen eines Reisestipendiums des *Tavistock Institutes* sämtliche Forschungsteams in den USA traf, darunter auch das Palo Alto-Team (Laing & Mullan 1995: 166).

Im Antrag vertrat Laing eine sehr pragmatische Definition von Schizophrenie, in welcher die Schizophrenie zwar pathologisch blieb, sich aber dem Bereich der „Normalität“ annäherte. Schizophrenie sei aus Perspektive der Phänomenologie und der interpersonalen Dynamik eine Lebensbedingung („condition of living“), bei der Menschen eine geringe Toleranz für schizogenen Stress hätten. Das heißt, aus dieser Perspektive sei Schizophrenie keine Ansammlung von Symptomen oder Störungen, sondern die Tendenz, die „persönliche Einheit“, die Einheit der Wahrnehmung und des Handelns ab einem bestimmten Stresspegel zu verlieren (Draft Application 1960: 14). Das Auslösen eines schizophrenen Zustands nannte Laing „Schizogenese“: Eine Person oder ein Netzwerk von Personen könne bewusst oder unbewusst, willkürlich oder unwillkürlich so handeln, dass die Fähigkeit einer anderen Person, frei zu handeln und ganzheitlich wahrzunehmen, oder ihre Fähigkeit oder Freiheit, sich selbst und andere Personen als „Person“ wahrzunehmen, beeinträchtigt werde (ebd.: 13–14).

Das gesellschaftliche Interesse, das in Laings und Estersons Schizophrenieverständnis und in ihrer Fragestellung angelegt war, stand also einerseits in der Tradition der amerikanischen Schizophrenieforschung der 1950er Jahre und war andererseits schon von Bleulers Definition der Schizophrenie als „Assoziationsstörung“ beeinflusst. Die Anordnung ihres Forschungsprojektes glich der amerikanischen, beruhte wie diese auf der Annahme, dass es „schizogene“ Familienumgebungen gab, und hatte dasselbe Erkenntnisinteresse: herauszufinden, wie Schizophrenie ausgelöst wurde, und welche Rolle dabei die Familie spielte. Zu diesem Zeitpunkt führte dieses Erkenntnisinteresse sie aber noch nicht dazu, die Gesellschaft grundsätzlich zu hinterfragen. Laings phänomenologisches Schizophrenieverständnis, das Schizophrenie weniger als Pathologie denn als Lebensumstand konzeptualisierte, stellte außerdem die grundsätzliche Existenzberechtigung der Schizophreniediagnose nicht in Frage. Diese Feststellungen sind deshalb bemerkenswert, weil sich Laing und Esterson in der aus dem Projekt resultierenden Publikation *Sanity, Madness and the Family *vom Vorhaben, eine Ätiologie der Schizophrenie herzuleiten, umfassend distanzierten: In erster Linie deshalb, weil sie darin die Berechtigung einer Schizophreniediagnose radikal hinterfragten und infolgedessen auch die Frage aufwarfen, wer „kränker“ sei: die vermeintlich Schizophrenen oder aber die Gesellschaft, die für diesen Zustand verantwortlich war.

## Die Banalität des Normalen

Im April 1962 begannen Laing und Esterson „normale“ Familien in ihre Forschung einzubeziehen, obschon Laing im Antrag 1960 den Nutzen einer Kontrollgruppe noch nicht gesehen hatte (ebd.: 29).^12^ Ziel war es einerseits, einen Kontrast zu den Familien mit Schizophrenen herzustellen. Andererseits wollten Laing und Esterson eine Art allgemeine Typologie von Familien ausarbeiten, wie Laing in einem Antrag um weitere Gelder schrieb.^13^

Die Ergebnisse dieser Forschung mit „normalen“ Familien sollten ursprünglich als zweiter Band, nach *Sanity, Madness and the Family. Volume I: Families of Schizophrenics* publiziert werden.^14^ Dieser Band erschien allerdings nie: Der Zusatz „Volume I“ fehlte dementsprechend ab der zweiten Auflage 1969. Weshalb die Publikation ausblieb, und was die Ergebnisse der Forschung waren, ist unklar.^15^ Die Auseinandersetzung mit diesen Fragen ist aber insofern interessant, als sich hier Ambivalenzen und Widersprüchlichkeiten zeigen, die, wie ich ausführen werde, mit den Grenzen der Vereinbarkeit von sozialkritischer Intervention und den ökonomischen und institutionellen Zwängen wissenschaftlicher Forschung zusammenhingen.

Laing selbst behauptete 1995 gegenüber dem britischen Autor Bob Mullan, die „normalen“ Familien seien schrecklich langweilig und eben normal gewesen (274):

It was like Samuel Beckett, reams and reams and reams of nothing. […] I thought, how was I going to dress it up to publish it? I really couldn’t publish a whole book of transcripts of people talking about margarine or fuck all. I could take on a particular family and characterize it but there was nothing really to characterize (ebd.: 281).

Die Familien, so Laing in der Rückschau, seien so angepasst und belanglos gewesen, dass er seine Leser*innen nicht damit habe langweilen wollen. Auf Nachfrage von Mullan präzisierte Laing, der Verzicht auf eine Publikation sei auch seiner privaten Situation geschuldet gewesen. Außerdem sei es ihm nicht gelungen, eine kohärente, systematische theoretische Analyse von Familiensystemen und deren Verwicklung in weiteren Netzwerken zu bewerkstelligen, die besser sei, als jene, die es zu diesem Zeitpunkt schon gab (ebd.: 283–284).^16^ In seiner Laing-Biographie erklärt Kotowicz (1997: 47) das Fehlen des zweiten Bandes von *Sanity, Madness and the Family* damit, dass Laing sich wohl nie wirklich für „Normalität“ interessiert habe. Ich sehe eine weitere Erklärung: Laing verlor das Interesse für die Fertigstellung des zweiten Bandes, weil er nach dem Erscheinen des ersten Bandes aufgrund verschiedener Faktoren einen Weg einschlug, der ihn von der akademischen Forschung weg – und stärker in die Öffentlichkeit und in alternative Foren führte, von wo aus er Psychiatrie und Gesellschaft gleichermaßen anprangerte.

1965 äußerte sich Laing noch optimistisch hinsichtlich der Ergebnisse der Kontrollstudie: „Preliminary findings seem to show that our indices do discriminate quite sharply between the families of schizophrenics and others“.^17^ Nur ein Jahr später stellte Laing seine Resultate im Rahmen einer Tagung zum Thema „Society and Psychosis“ in Philadelphia anders dar. Die Tagung war vom Psychiater Ross Speck organisiert worden, der ebenfalls zu Schizophrenie in Familien forschte, und unter den Teilnehmenden befanden sich weitere Familienforschende wie auch Gregory Bateson. Die Tagung verfolgte die Leitfrage: „If the whole world has gone mad, how can we expect the individual to be sane?“^18^ Laing berichtete in diesem Rahmen, dass er seine Annahme, dass Eltern in „normalen“ Familien eine offene Kommunikation zu ihren Kindern pflegten, habe revidieren müssen. Seine Studie „normaler“ Familien habe gezeigt, dass diese Familien das Spiel der „Mystifizierung“ viel stärker mitspielten als angenommen.^19^ Das Konzept der „Mystifizierung“ stellten Laing und Esterson bei der Publikation ihrer Schizophrenieforschung vor. Es wies große Ähnlichkeiten zur „Double Bind“-Theorie des Bateson-Teams auf sowie zu anderen zeitgenössischen Konzepten wie etwa das „masking“ bei Theodore Lidz oder die Vorstellung von „sham“ beim Anthropologen Jules Henry. Gemeinsam war den Konzepten eine grundsätzliche Kritik an der mangelnden Authentizität der Familien. Wie der Historiker Jamie Cohen-Cole gezeigt hat, gehörte Authentizität im Kontrast zu Konformität zu den Eigenschaften, die amerikanische Sozialpsycholog*innen im Kalten Krieg als entscheidend für die Zukunft des Liberalismus und der Demokratie definierten (Cohen-Cole 2016: 36).

Ich möchte zwei Erklärungen für diese recht unterschiedlichen Deutungen der Forschung an „normalen“ Familien vorschlagen, welche die jeweiligen Publikationsorte berücksichtigen und einander somit nicht ausschließen.

1. Im Jahresbericht war es wichtig, die Resultate der Studie so darzustellen, wie sie zum einen erwartet wurden und zum andern die Forschung und ihre Fortsetzung am meisten legitimierten. Da Laing mit Geldern des amerikanischen *Foundations Fund for Research in Psychiatry* und des *Tavistock Institute* arbeitete, musste er Resultate vorhersagen, die den größten Nutzen für die Behandlung von Schizophrenie versprachen. Eine möglichst große Diskrepanz zwischen „gesunden“ und „pathogenen“ Familien war in diesem Zusammenhang das attraktivere Resultat, da eine Intervention bei den „pathogenen“ Familien einen therapeutischen Erfolg bei den Schizophrenen versprach. Die Feststellung, dass alle Familien gleichermaßen krankhafte Interaktionen aufwiesen, erschien hingegen als therapeutische Sackgasse und somit als ein unerwünschtes Forschungsresultat. Anders verhielt es sich bei der „Society and Psychosis“-Tagung, an der sich alle Teilnehmenden mehr oder weniger einig waren, dass mit der Gesellschaft an sich etwas nicht stimmte. Somit war das Setting für Laings Deutungen der Forschungsresultate und ihre Kommunikation entscheidend.
2. Besonders wenn Laings rückblickende Interpretation von 1995 einbezogen wird, können die unterschiedlichen Deutungen auch als zwei Seiten einer Medaille angesehen werden. Die dröge „Normalität“ der „normalen“ Familien beschrieb Laing 1995 als erstickend: Es gehe den Familien zwar nicht besonders schlecht, aber auch nicht besonders gut; jegliche Form von Ausbruch in die eine oder andere Richtung sei nicht zu erwarten. Insofern waren für ihn diese Familien gesellschaftlich gesehen auch pathogen, jedoch in einem akzeptierten Rahmen. Als Mullan Laing fragte, ob er nie etwas zu dieser Banalität des Normalen habe schreiben wollen, antwortete Laing: „What’s the alternative? State intervention? State planned social engineering to break up that family system and design another type where children are brought up not by their biological parents, but by some state appointees or experts in child rearing?“ (Laing & Mullan 1995: 283) David Cooper (1971) – ein anderer bekannter Vertreter der Antipsychiatrie-Bewegung und kurzzeitiger Weggefährte von Laing – möge den Tod der Familie verkündet haben, so bemerkte Laing, aber trotz Sympathien für diese Position fand er diese Form von Kritik zu wenig konstruktiv (Laing & Mullan 1995: 283). Mit anderen Worten: So lange sich an der Gesellschaftsform als Ganzes nichts änderte, waren diese „normalen“ Familien die adäquate Familienform; ihre Banalität war lediglich ein Symptom für die Gesamtgesellschaft. Somit wären sowohl die Aussage, dass „normale“ Familien normal seien, als auch die Aussage, dass „normale“ Familien pathogen seien, wahr. Familien Schizophrener überschritten in Laings und Estersons Darstellung gesellschaftlich akzeptierte Formen dieser krankhaften „Normalität“ und waren insofern anders als die „normalen“ Familien, gleichzeitig äußerte sich ihre Pathogenität, wie ich im Folgenden zeigen werde, durch eine Steigerung dieser „Normalität“ in eine übermäßige Angepasstheit.

Beide Erklärungen verdeutlichen, dass Laing sich hier an einem Wendepunkt befand. Als Angestellter des Tavistock Institutes und als auf Drittmittel angewiesener Forscher, war er bis Mitte der 1960er Jahre institutionellen Praktiken und Logiken unterworfen, welche eine bestimmte Form der Wissensproduktion begünstigten. Laings politische Überzeugungen sprengten diesen Rahmen zunehmend. Die Einsicht, dass „normale“ Familien ebenso gestört seien wie „kranke“, beziehungsweise dass die „Normalität“ an sich krankhaft sei, verlangte wiederum geradezu nach politischer Handlung und dem Bruch mit dem Establishment wie auch mit der Akademie. Die Position, die Laing 1995 gegenüber Mullan vertrat, illustriert zudem Laings Zugang zur Politik: Er lehnte jede Art (staatlicher) Herrschaft ab und strebte, wie ich weiter unten zeigen werde, gesellschaftlichen Wandel durch die Ermöglichung einer Bewusstseinsänderung beziehungsweise eines „wahren“ Bewusstseins an.

## Von der Schizophrenie zur „Schizophrenie“: Das Buch *Sanity, Madness and the Family*

1964 erschien schließlich das Buch *Sanity, Madness and the Family* – zunächst im Hausverlag des *Tavistock Institute*.^20^ Das Endprodukt der Forschung Laings und Estersons wies einige Unterschiede zu den im Antrag formulierten Forschungszielen auf und zeigt – obwohl es sich damit immer noch um einen wissenschaftlichen Beitrag handelte und auch so rezipiert wurde – wie Laings Denken zunehmend inkompatibel mit der etablierten Psychiatrie wurde und eine gewisse Sprengkraft entwickelte.

Bereits im Antrag hatte Laing Schizophrenie nicht als Krankheit, sondern als Lebensbedingung definiert. In der Einleitung zu *Sanity, Madness and the Family* ging er mit Esterson noch einen Schritt weiter: „We shall use the expression ‚schizophrenic‘ for a person or for his experience or behavior in so far as he, his experience, or his behavior, are clinically regarded as betokening the presence of ‚schizophrenia‘“ (Laing & Esterson 1972: 17). Den Begriff „Schizophrenie“ und alle davon abgeleiteten Begriffe setzten sie durchwegs in Anführungszeichen und erklärten, dass sie den Begriff lediglich pragmatisch einsetzten. Das heißt, sie verwendeten ihn, um die realen sozialen Effekte zu beschreiben, welche die Zuschreibung als „schizophren“ hatten. Die Frage, ob es Schizophrenie gäbe, und ob die Patientinnen in ihren Berichten tatsächlich schizophren seien, würden sie bewusst ausklammern, so Laing und Esterson (ebd.: 19). Trotz dieser in ihrer Begrifflichkeit „agnostischen“ Position stellten sie damit die Existenz von Schizophrenie als organische oder psychische Krankheit zumindest in Frage. Dies hatte zur Folge, dass ihr Buch auch keinen Anspruch darauf erhob, einen Beitrag zur Ätiologie der Schizophrenie zu leisten, anders als dies im Antrag noch der Fall gewesen war. Der ganze Aspekt der „Schizogenese“ fehlte konsequenterweise in der Buchversion ihrer Untersuchung. Dennoch sah Laing – wie er anderswo beschrieb – das Ergebnis ihrer Forschung darin, dass es ihnen gelungen sei zu zeigen, dass die als „Schizophrenie“ beschriebenen Verhaltensweisen jeweils eine direkte Folge des sozialen Subsystems der Familie seien.^21^ Auch wenn *Sanity, Madness and the Family* also nicht den Anspruch erhob, ätiologische Thesen im engeren Sinne aufzustellen, so sah Laing darin dennoch bewiesen, dass der soziale Zustand, der unter „Schizophrenie“ gefasst wurde, eine Fortschreibung der Familiendynamik darstellte und insofern durch gesellschaftliche Verhältnisse hervorgebracht wurde. Indem Laing und Esterson das Buch nicht als ätiologische Studie rahmten, Schizophrenie als Diagnose in Frage stellten und das unter „Schizophrenie“ gefasste Verhalten als sozial erklärbar präsentierten, positionierten sie das Buch – möglicherweise ungewollt – außerhalb des psychiatrischen Mainstreams und nahmen im Unterschied zum Antrag 1960 nun eine gesellschafts- und psychiatriekritischere Haltung ein.

*Sanity, Madness and the Family* besteht aus einer theoretisch-methodischen Einleitung und elf Kapiteln, in welchen je eine Familie detailliert beschrieben wird. Es handelt sich dabei um romanartige Erzählungen; ab und zu wird der Originalton einer Interaktion wiedergegeben oder eine Tabelle als Illustration verwendet, selten ziehen die Autoren zwischendurch ein Fazit oder setzen eine Fußnote mit einer weiterführenden Interpretation. Kotowicz (1997: 46) beschreibt die Kapitel als „pictures of devastation“. Ziel dieser Beschreibungen, so Laing und Esterson (1972: 27), sei es gewesen, zu zeigen, dass jene Verhaltensweisen, welche von den Familien und dem klinischen Personal als Schizophreniesymptome wahrgenommen wurden, aus ihrem Kontext nachvollziehbar seien.

Die im Buch beschriebenen Eltern hegten stets den Wunsch nach Konformität, dem die als „schizophren“ Diagnostizierten nicht entsprechen konnten oder wollten. Die Eltern hatten außerdem oft rigide Vorstellungen davon, wie ihre Kinder sein sollten und verleugneten häufig Dinge, die sie nicht wahrhaben wollten und die sie überforderten, und „mystifizierten“ dadurch ihre Kinder. Die in *Sanity, Madness and the Family* beschriebenen jungen Frauen gerieten deshalb in Lebensumstände, die Laing und Esterson als unhaltbare Situationen („untenable positions“) beschrieben, weil ihr Streben nach Authentizität und Freiheit im Vergleich zu den Erwachsenen noch nicht komplett gebrochen war. Sie erschienen im Buch als unschuldige Subjekte, die von ihren repressiven und verlogenen Familien manipuliert wurden. Anstatt sich anzupassen und die verlogene Wirklichkeit ihrer Familien zu akzeptieren, gingen diese jungen Frauen den einzigen Weg des Widerstands, der sich ihnen eröffnete: entweder den totalen Rückzug oder Rebellion; beides brachte ihnen eine psychiatrische Diagnose ein. Dies lässt sich etwa am Beispiel des Kapitels zur Familie Field illustrieren: Als June nach einer Phase des Rückzugs wieder aufzuleben begann und ihre Meinung über ihre Mutter, die Schule, Gott und andere Menschen kundtat – wenn auch, wie Laing und Esterson fanden, ziemlich kleinlaut –, wurde sie von ihrer Mutter als krank und nicht mehr „sie selbst“ angesehen. Junes Eltern wirkten Laings und Estersons Beschreibung zufolge mystifizierend, indem sie Junes „wirkliches autonomes Selbst“ als Krankheit definierten. Dadurch versuchten sie unter dem Deckmantel der Sorge um ihre psychische Gesundheit ihre Selbstentfaltung zu unterbinden und sie in ihrer Abhängigkeit zu wahren.

Die Motive, die in *Sanity, Madnes and the Family *verhandelt werden – Entfremdung, Autonomiestreben, Nonkonformismus, Sexualität und versuchte Selbstverwirklichung Jugendlicher in einer in Zwängen und Tabus erstarrten bürgerlichen Gesellschaft – waren auch zentral für die sich in den 1960er Jahren formierende gegenkulturelle Jugendbewegung. Außerdem basierte Laings und Estersons Interpretationsmuster auf einer erweiterten Form der in Freuds Kulturtheorie entwickelten „Repressionshypothese“, welche in den 1960er Jahren zum Beispiel durch die Schriften Wilhelm Reichs und Herbert Marcuses in links-intellektuellen Kreisen starke Verbreitung fand. Im Unterschied zur amerikanischen Schizophrenie-Forschung stand das Wissen über Schizophrenie und Familie, das aus Laings und Estersons Forschung hervorging, in einem direkten Dialog zum vorhandenen gesellschaftlichen Unbehagen und griff zudem Theorien auf, die bereits einer explizit politischen Ausrichtung ihrer Forschung zuzuordnen waren. Auf beides werde ich im nächsten Abschnitt näher eingehen.

Diese gesellschaftliche Dimension und radikalere Sichtweise, die in* Sanity, Madness and the Family* implizit und bisweilen auch explizit entwickelt wurden, fanden indes in den Rezensionen kaum Beachtung.^22^ Stattdessen wurde die Erstauflage des Werks so gelesen wie die Publikationen aus der konventionellen Psychiatrie, an die es anknüpfte. Kritisiert wurde in erster Linie die wissenschaftliche Methode, wie beispielsweise das Fehlen einer Kontrollgruppe. Das Buch war auch, trotz der Distanzierung zur Mainstream-Psychiatrie und obwohl es über Fachgrenzen hinweg rezipiert wurde, in erster Linie noch an ein wissenschaftliches Publikum adressiert. Dies änderte sich, wie ich im folgenden Kapitel zeigen werde, zusehends mit Laings nachfolgenden Publikationen sowie mit den weiteren Auflagen seiner bereits publizierten Werke. Dies lag einerseits an den Medien selbst: Laing publizierte in populäreren Medien und seine Bücher wurden als Taschenbücher bei Penguin statt in akademischen Verlagen (neu) aufgelegt. Andererseits spitzte sich seine radikale Sichtweise und gesellschaftliche Positionierung weiter zu und war nicht mehr zu ignorieren.^23^

## Schizophrenie als Durchbruch. Laings Weiterentwicklung und die Politisierung seines Schizophreniebegriffs

1964, im Jahr der Erstpublikation von *Sanity, Madness and the Family,* sang Bob Dylan „The Times They Are A‑Changin’“. Der Cannabis-Konsum nahm markant zu, LSD verließ die Klinik und wurde für die bewegte Jugend zu einem beliebten Mittel der Selbsterfahrung, die Anti-Baby-Pille setzte sich durch (Tanner 1998: 210): Es herrschte Aufbruchsstimmung. Auch der eigentliche Beginn der sogenannten Anti-Psychiatrie wird von Psychiatriehistoriker*innen auf das Jahr 1964 datiert (Wall 2017: 122; Crossley 1998: 886): In diesem Schlüsseljahr für die Gegenkultur der Sechzigerjahre begannen auch viele Exponenten der Antipsychiatrie sich erstmals politisch zu äußern und zu engagieren. Für Laing war 1964 ein äußerst produktives Jahr. In seinen zahlreichen Publikationen, die im Verlauf des Jahres erschienen, lässt sich im Vergleich zu den vorherigen eher akademisch ausgerichteten Publikationen eine dezidiert psychiatrie- und gesellschaftskritische Position erkennen sowie damit einhergehend eine zunehmende Diversifizierung seiner Adressat*innen. Sedgwick bezeichnete diese Phase Laings als seinen „radical trip“ (Sedgwick 1982: 66).

Für *New Society*, ein neu gegründetes, politisch unabhängiges Magazin, verfasste Laing (1964a) einen Artikel, in dem er die Theorien vorstellte, die ihn in den letzten Jahren am stärksten geprägt hatten: Szasz’ Kritik an psychiatrischen Diagnosen aus *The Myth of Mental Illness*, Goffmans Labeling-Theorie aus *Asylums* und die Double Bind-Theorie aus „Toward a Theory of Schizophrenia“ von Bateson und seinen Mitarbeitern. Diese Texte, befand Laing, seien Einblicke in eine Revolution der Konzeption von Wahnsinn und psychischer Gesundheit, die gerade innerhalb und außerhalb der Psychiatrie stattfände. An ein akademisches Publikum gerichtet erklärte Laing (1964b: 193) im selben Jahr im *International Journal of Social Psychiatry,* basierend auf ebendiesen Theorien, weshalb der Schizophreniebegriff grundsätzlich hinterfragt werden müsse. Ähnlich wie in *Sanity, Madness and the Family* argumentierte Laing, Schizophrenie könne nicht als Krankheit verstanden, sondern müsse als Label für eine bestimmte Art eigenartigen Verhaltens gesehen werden (ebd.: 186). Dies führte ihn zu einer grundsätzlichen Kritik dessen, was im Bereich psychischer Erfahrung als Norm gelten könne. Man müsse die Annahme überwinden, dass Schizophrene von der Realität entfremdeter seien als man selbst, und die eigene Norm als Maßstab für psychische Gesundheit hinterfragen (ebd.: 190).

Dabei ging es Laing nicht um eine rein theoretische Auseinandersetzung. Dieses Umdenken forderte er auch deshalb von seinen Fachkolleg*innen, weil er sich davon einen in seinen Worten „humaneren“ Umgang mit Schizophrenen erhoffte. Er plädierte für die Installierung therapeutischer Gemeinschaften und Akutzentren, in denen „Schizophrene“ auf ganzheitliche und respektvolle Weise auch von Menschen begleitet würden, die ähnliche Erfahrungen gemacht hätten wie sie, und nicht (nur) von Psychiater*innen (ebd.: 192–193). Laings Artikel ist eine Auseinandersetzung mit der Psychiatrie, die sich an seine *scientific community* richtet und sowohl einen Perspektivenwechsel als auch eine Revolution der psychiatrischen Praxis fordert. Insofern kann Laings Artikel durchaus als politische Intervention innerhalb der Psychiatrie gelesen werden. Umso mehr als Überlegungen wie diese und die daraus resultierenden Experimente schließlich zur Deinstitutionalisierung der Psychiatrie in Großbritannien beitragen sollten (Mc Geachan 2016: 212). Laing selbst sollte bereits 1965 mit Kingsley Hall einen Teil seiner hier formulierten theoretischen Forderungen in die Tat umsetzen.

Was Laing genau mit dem Zustand der „Entfremdung“ meinte, der in der Psychiatrie bisher als Norm gegolten habe, offenbart sich in einem weiteren Text von 1964, seiner Einleitung zur Neuauflage von *The Divided Self*:

A man who prefers to be dead rather than Red is normal. A man who says he has lost his soul is mad. A man who says that men are machines may be a great scientist. A man who says he is a machine is ‚depersonalized‘ in psychiatric jargon. A man who says that Negroes are an inferior race may be widely respected. A man who says his whiteness is a form of cancer is certifiable. A little girl of seventeen in a mental hospital told me she was terrified because the Atom Bomb was inside her. That is a delusion. The statesmen of the world who boast and threaten that they have Doomsday weapons are far more dangerous, and far more estranged from ‚reality‘ than many of the people on whom the label ‚psychotic‘ is affixed (Laing 1990: 11–12).^24^

Laing stellte in diesem Zitat gesellschaftlich sanktionierte Wahnvorstellungen von Psychotiker*innen radikalem Antikommunismus, Rassismus und der Bedrohung eines Atomkriegs gegenüber, die eine weit größere Bedrohung darstellten, jedoch gesellschaftlich akzeptiert seien. Damit hinterfragte er den Bezugsrahmen, an dem gemessen wurde, was „normal“ und was „wahnsinnig“ ist. Die Wahnvorstellungen der Psychotiker*innen, so glaubte Laing, würden im Kontext des „normalen“ Wahnsinns als normal erscheinen oder sogar als normaler, weil sie den realen Wahnsinn nicht verdrängten, sondern ihm einen durchaus adäquaten Ausdruck gäben.

Laings Kritik an einer entfremdeten Gesellschaft, die ihren eigenen Wahnsinn nicht erkenne, fügte sich nahtlos sowohl in eine freudomarxistische als auch eine existentialistische Gesellschaftskritik der 1960er Jahre ein. Laing bezog sich insbesondere auf Marcuses *One-Dimensional Man* sowie auf Jean-Paul Sartre. Ersteres Werk hatte Laing gerade für die *New Left Review* rezensiert (1964c) und zusammen mit David Cooper hatte er Sartres gesamtes bisheriges Œuvre, in dessen Zentrum eine existentialistische Entfremdungstheorie steht, unter dem Titel *Reason and Violence* für englischsprachige Leser*innen zusammengefasst (Laing & Cooper 1964).^25^ Wie Sartre und Marcuse glaubte Laing, dass die Menschen in einer entfremdeten Gesellschaft ihre verdinglichte Lebenswelt verinnerlichen. Die verinnerlichte Außenwelt ersetzte das authentische Selbst, von dem sich die Menschen entfremdet hätten (DeKoven 2004: 202). Laing, so zeigt sich in der neuen Einleitung zu *The Divided Self,* schloss an die freudomarxistische Kulturtheorie an, welche die kulturelle Triebunterdrückung als Quelle von Leid identifizierte. Diese Repressionshypothese weitete Laing aber aus: Nicht nur die menschlichen „Instinkte“ und nicht nur die Sexualität würden von der Gesellschaft unterdrückt, sondern jegliche transzendentale Erfahrung:

Among one-dimensional men, it is not surprising that someone with an insistent experience of other dimensions, that he cannot entirely deny or forget, will run the risk either of being destroyed by the others, or of betraying what he knows (Laing 1990: 11).^26^

Die Bedeutung der Erfahrung in Laings Argumentation zeigt den Einfluss Sartres auf seine Freud-Rezeption. Was er mit transzendentalen Erfahrungen meinte, erschließt sich in einem Artikel für die *New Left Review* mit dem Titel „What Is Schizophrenia?“.^27^ Laing (1964d: 65–66) vertrat darin die Ansicht, dass manche als schizophren bezeichnete Menschen außergewöhnliche Erfahrungen hätten, die eigentlich Teil einer planmäßigen, natürlichen Sequenz von Erfahrungen seien. Es handle sich bei dieser Sequenz um eine Reise ins Innere, eine Reise zum Selbst. Diese innere Reise sei jedoch gesellschaftlich nicht akzeptiert und werde als pathologisch betrachtet. Anstatt diese abzuwerten und die Reisenden in Anstalten einzuweisen, sollten sie durch eine Begleitung und ein Initiationsritual ermutigt werden, plädierte Laing (ebd.: 67). Das Abspielen dieser natürlichen Sequenz, so schloss er seine Überlegungen, sei der Kern einer wirklich gesunden („truly sane“) Gesellschaft. Falls die Menschheit überleben sollte, werde sie auf die sogenannte Zeit der Aufklärung als eine Zeit der Dunkelheit zurückblicken: „They will see what we call ‚schizophrenia‘ was one of the forms in which, often through quite ordinary people, the light began to break through the cracks in our all-too-closed minds“ (ebd.: 68).

Laing richtete sich mit diesem Artikel an ein breites Publikum des linken politischen Spektrums. Die *New Left Review *war wie *New Society *ebenfalls ein sehr junges Magazin. Es war 1960 von Stuart Hall gegründet worden und sollte zu einer der einflussreichsten Publikationen einer Neuen Linken in Großbritannien (und darüber hinaus) avancieren. Laing beschrieb in seinem Artikel Schizophrene als Menschen, die – in einer Gesellschaft, die jegliche transzendentale Erfahrung unterdrücke und von ihrem authentischen Selbst entfremdet sei – als Einzige Zugang zu einem wahren Bewusstsein hätten. Somit stellten sie den Schlüssel zu einer gesunden Gesellschaft dar.

Der deutsche linksradikale Psychotherapeut Jörg Bopp (1980) kritisierte Laing dafür, dass seinem Begriff der Entfremdung die sozio-ökonomische Dimension fehle, und er auf die „Demolierung der inneren Welt“ begrenzt sei (49). Diese Kritik verdeutlicht, dass Laings politische Auffassung von derjenigen seiner marxistischeren Kolleg*innen wie beispielsweise Cooper abwich. Hingegen lassen sich in der Vorstellung, dass der Mensch sein Potential aufgrund der Entfremdung durch die Gesellschaft nicht voll entfalten könne und dass seine Befreiung in der Zuwendung zum Selbst und in einer radikalen Subjektivität zu suchen sei, Parallelen zum (neuen) New Age-Diskurs feststellen, wie er sich in Großbritannien und anderswo in den 1960er Jahren zu verbreiten begann (Miller 2012: 145).^28^ So schreibt auch Bopp (1980: 50), in Laings Vorstellung von transzendentaler Erfahrung würden sich „Traditionen abendländischer Mystik, romantische Selbstversenkung und Naturerfahrung“ mit „psychedelischen Erlebnisqualitäten und Frömmigkeitsformen aus ostasiatischen Religionen“ vermischen.

Mit der radikal positiven Umwertung der Schizophrenie und Laings Fokus auf die Befreiung des Selbst wich seine Position jetzt aber auf jeden Fall sehr deutlich von der seiner amerikanischen Kollegen ab. Laing unterschied sich aber auch insofern von anderen Schizophrenie-Forschenden, als dass ihn seine Forschungsfrage über das Familienumfeld hinaus auf eine politische Ebene führte: Der Bezugsrahmen seiner Fragen hatte sich verschoben (Crossley 2006: 115). In seinem Artikel für die *New Left Review* forderte er, dass sich die Schizophrenieforschung von der Analyse gestörter Familienbeziehungen und -kommunikation auf die nächsthöhere Ebene begeben müsse: die Ebene der Zivilgesellschaft. Es gelte die politische Ordnung zu verstehen, die Art und Weise, in der Menschen Herrschaft und Macht übereinander ausüben würden (Laing 1964d: 65).

Laings Publikationen aus dem Jahr 1964 zeigen, dass das Feld der akademischen Psychiatrie für Laing zu eng geworden war. Seine Erkenntnisse aus seiner Forschung und seiner Arbeit mit „Schizophrenen“ wiesen weit über dieses Feld hinaus und mussten entsprechend anders kommuniziert werden. Außerdem wird deutlich, wie Laing zunehmend aktiv und passiv in die Gegenkultur der 1960er Jahre eingebunden war.

## Laing als Groovy Scientist und politischer Akteur

Laings radikale Auslegung der freudschen Kulturtheorie und seine Beschreibung der Schizophrenie als transzendentale Erfahrung ebneten ihm den Weg für ein neues Publikum jenseits des wissenschaftlichen Fachkreises. Der Psychoanalytiker Peter Mezan (1972) beschreibt in einem Artikel für *Esquire* Laings Publikum in den späten 1960er Jahren als schlendernde Studierende, in deren Hosentaschen neue Exemplare von *The Divided Self* oder *The Politics of Experience and The Bird of Paradise* steckten. Diese Bücher, so Mezan, sahen sie als Bestätigung dafür, dass Wahnsinn ein legitimer Zustand sein könne, „a place one could legitimately go should things get any worse, a high-class resort where many of one’s friends held advance reservations“. In dieser etwas verkürzten Leseart Laings wurde Wahnsinn als politischer Befreiungsschlag, als revolutionärer Akt in einer gleichgeschalteten Massengesellschaft überhöht. Wie die Kulturwissenschaftlerin Marianne DeKoven (2004: 203) feststellt, spiegelten Laings Schriften ab Mitte der 1960er Jahre das Gefühl der Gegenkultur jener Zeit, nämlich, dass die bestehenden Verhältnisse unerträglich seien und komplett verändert werden müssten. Dies nicht nur, damit die Menschheit ihr authentisches Potential befreien, sondern auch, damit das Leben überhaupt auf sinnhafte Weise weitergehen könne. Die Anschlussfähigkeit seiner Ideen zur Gegenkultur und seine Radikalisierung führten Laing im Verlauf der 1960er Jahre immer mehr zum politischen Aktivismus. Er vertrat seine Ansichten nun nicht mehr primär vor Fachkreisen, sondern trug seine Überzeugungen stärker in eine links-intellektuelle Öffentlichkeit.

Als 1967 ein schmales Büchlein namens *The Politics of Experience and The Bird of Paradise* erschien, hatte sich Laings Wahrnehmung in der Öffentlichkeit endgültig geändert (Laing 1967). Laut Kotowicz (1997: 3) sei er durch diese Publikation zu einer Art eigenwilligem Guru der Schizophrenen geworden (ebd.). Außerdem begann das Gerücht zu kursieren, Laing habe selbst den Verstand verloren und sei in eine Anstalt eingewiesen worden (Mezan 1972), was auf pointierte Weise zeigt, dass seine Aussagen und Texte nicht mehr als psychiatrische Fachliteratur akzeptiert wurden. Tatsächlich erzählte Laing in einem Interview, er habe gehört, dass manche Psychiater versucht hätten, ihn auf Basis seiner Bücher zu diagnostizieren (ebd.). Die im 1976 erschienenen Werk vertretenen Ansichten weichen jedoch nicht signifikant von denjenigen ab, die Laing schon 1964 vertreten hatte. Das Büchlein enthielt in erster Linie eine erweiterte und kombinierte Fassung aller drei Artikel über Schizophrenie von 1964 zusammen mit weiteren Texten, welche das Verhältnis von Wahnsinn und „Normalität“ auf den Kopf stellten und von der Entfremdung vom und der Suche nach einem authentischen, inneren Selbst sowie nach einer transzendentalen Wahrheit handelten. Was sich verändert hatte, war in erster Linie der Rezeptionskontext. Einerseits beschleunigte sich in den Jahren zwischen 1964 und 1967 der gesellschaftliche Wandel, was dazu führte, dass die Psychiatriekritik einem größeren Publikum zuteilwurde (Majerus 2010; Tanner 1998: 216), nachdem sie sich in Europa und den USA seit den späten 1950er Jahren zu verbreiten begonnen hatte. Andererseits adressierte Laing nun kein wissenschaftliches Publikum mehr, sondern direkt die Vertreter*innen einer intellektuellen Gegenkultur (Harrington 2012: 1292). Er nahm eine neue Position als Anti-Psychiater und – Kaisers und McCrays Konzept folgend – als „Groovy Scientist“ ein.

Laings bereitwillige Annahme dieser neuen Rolle hatte mit verschiedenen Push- und Pull-Faktoren zu tun. Zu den Push-Faktoren gehörte seine zunehmend marginale Stellung in der Wissenschaft. So versuchte Laing seit 1962 einen Artikel im *British Journal of Psychiatry* zu veröffentlichen, mehrere Fassungen wurden jedoch abgelehnt. Crossley (1998: 882) sieht dies als Zeichen dafür, dass Laing innerhalb der Wissenschaft immer stärker marginalisiert wurde und er sich schließlich zurückzog. Für diese Deutung spricht auch, dass Laings Vertrag mit dem *Tavistock Institute* 1966 auslief und er fortan in seiner Privatpraxis arbeitete, die er seit Anfang der 1960er Jahre betrieb. Wall (2017: 11) sieht allerdings keinen klaren Bruch zur konventionellen Psychiatrie. So sei Laing 1966 von Donald W. Winicott dringend dazu eingeladen worden, der *British Psychoanalytical Society* beizutreten und 1968 war Laing Co-Präsident des *Second Congress of Social Psychiatry*. Laing scheint also in den entsprechenden Fachgesellschaften durchaus noch Glaubwürdigkeit als Psychiater und Wissenschaftler gehabt zu haben. Eindeutiger sind die Pull-Faktoren zu identifizieren: In der Gegenkultur der 1960er Jahre erwiesen sich Laings Ideen als äußerst anschlussfähig und er stieß auf viele offene Ohren. Außerdem hatte Laing selbst eine Faszination für die berühmten Exponenten der Gegenkultur. So reiste er 1964 ein zweites Mal in die USA, wo er unter anderen dem Harvard-Psychologen und LSD-Guru Timothy Leary und Allen Ginsberg begegnete (Crossley 1998: 885). In der Laing gewidmeten Sonderausgabe des New Yorker Kulturmagazins *Salmagundi *von 1971 schrieb der Herausgeber, Laing habe sich einen Ruf als Psychiater erarbeitet, der Schizophrene behandle, aber sein heutiges Publikum habe nur wenig Ahnung von psychischer Krankheit oder Psychoanalyse. „[I]t is as a culture critic, a new species of the psychiatrist as a prophet, that Laing would be known […]“ (Boyers 1971: 3–4). Damit beschreibt er Laings neue Position als „Groovy Scientist“, als populärer und unkonventioneller Wissenschaftler, der gleichzeitig auch mit seiner gesellschaftspolitischen Positionierung außergewöhnliche Verbreitung fand. Weil Laing die Analyse psychischer Krankheiten mit einem Angriff auf das Fundament der westlichen Zivilisation verbinde, so der Herausgeber weiter, habe er sowohl Philosoph*innen, Soziolog*innen, Literat*innen, Religionswissenschaftler*innen sowie jede Gruppe von Menschen angesprochen, die sich für die Ideengeschichte und die gegenwärtige Kultur interessierten – mit Ausnahme der Kolleg*innen aus der Psychiatrie (ebd.).

## Treiber der Anti-Psychiatrie

Laings Neuausrichtung zeigte sich nicht nur im Wechsel der bevorzugten Publikationsorgane und seiner Adressaten, sondern auch in seinem neuen Wirkungsfeld. Er war zunehmend aktiv an der Gestaltung gegenkultureller Einrichtungen beteiligt. 1965 gründete Laing zusammen mit Aaron Esterson, David Cooper und anderen die Organisation „Philadelphia Association“ mit der sie eine therapeutische Gemeinschaft in Kingsley Hall, einem alten Gebäude im Londoner East End, betrieben. Kingsley Hall war mit den Worten Coopers, der mit der „Villa 21“ bereits ein ähnliches Experiment gewagt hatte, ein „anti-hospital“ (Wall 2013). In einem solchen existieren – zumindest theoretisch – keine Hierarchien zwischen Patient*innen und medizinisch Ausgebildeten, und es gibt keine Zwangseinweisungen oder -maßnahmen. Hier ist es allen gestattet, sich auf ihre Reise ins Selbst zu begeben, ohne durch therapeutische Interventionen gestört zu werden (Crossley 1999: 810). Kingsley Hall war jedoch mehr als eine therapeutische Gemeinschaft. Es wurden dort Seminare zu Themen wie Devianz, Psychiatriekritik, dem Double Bind, Familienstudien und Psychiatriegeschichte abgehalten. Dazu gab es Aktivitäten wie Malen, Weben, Yoga, Rezitationen von Gedichten, Indischer Tempeltanz, Ausstellungen, Filmprojektionen usw. (Laing 1999: 59–60). Außerdem waren viele Prominente hier zu Gast (Paton 2015). Kingsley Hall war also ein Treffpunkt der Londoner Gegenkultur, aber auch ein Ort, an dem Laings Forschung und Theorie eine praktische Anwendung erhielten. Darüber hinaus war Kingsley Hall auch eine politische Intervention, da Laing hier exemplarisch zeigen konnte, wie die Gesellschaft mit vermeintlich „Schizophrenen“ umgehen sollte. Er schuf damit auch einen utopischen Raum, in dem ein alternatives Zusammenleben möglich war. Es handelte sich dabei um ein soziales Experiment, das typisch für die 1960er Jahre war. Crossley (1999) spricht von Kingsley Hall als einer „working utopia“ – ein utopisches Projekt, das immer eine realitätsferne Dimension behielt, da seine Ziele nie vollständig verwirklicht werden konnten.

Auch die eingangs erwähnte Dialectics of Liberation-Tagung ist ein Ereignis, dass die politische Mobilisierung von Laings Theorien und die Verschmelzung des Wirkens der Anti-Psychiater mit der Gegenkultur aufzeigt. Das Flugblatt für den Kongress kündigte das Thema an:

A reign of terror is now perpetrated and perpetuated on a global scale. In the affluent societies, it is masked. There, children are conditioned by violence called love to assume their position as the would-be inheritors of the fruits of the earth. But, in the process, they are reduced to little more than hypothetical points on a dehumanized co-ordinate system.^29^

Laings Theorie, dass Kinder durch ihre Familien in eine entfremdete bürgerliche Ordnung überführt würden, erschien hier losgelöst von ihrem Bezug auf Schizophrenie, dafür mit apokalyptischer Dringlichkeit. Am Kongress herrschte keine Einigkeit, weder in Bezug auf ein geteiltes Vokabular, noch darüber, wie eine „Dialektik der Befreiung“ genau aussehen sollte (Jervis 1969: 7). Vielmehr zeigten sich auch bei diesem Ereignis die vielen gegensätzlichen Ideen und Stoßrichtungen der Gegenkultur, die vom Marxismus verschiedenster Prägung über New Age, Psychoanalyse bis hin zum Existentialismus inspiriert waren und in deren Tradition sich Laing und die Anti-Psychiatrie einfügten. Der italienische Antipsychiater Giovanni Jervis beschrieb im Tagungsband, der Kongress habe zunächst auf ihn gewirkt wie eine „Hippie-Folklore-Vorstellung“: „Von der Galerie aus regnete es Seifenblasen, Flötentöne waren zu hören, Manifeste mit provozierenden Slogans oder rotbeschriebene Klosettpapierbänder wurden herabgelassen; sogar lange Seile, an denen eine Schaukel hing.“ Jedoch sei es eben auch eine politische Veranstaltung gewesen, wie sich bei näherem Hinsehen offenbarte: „[D]er langhaarige Junge, der sich auf der Schaukel hin- und herschwang, erwies sich als ein politisch recht aktiver Student der London School of Economics, und die Slogans bezogen sich zuweilen sehr genau auf Worte des Vorsitzenden Mao“ (ebd.).

## Symbolbild des Aufbruchs

Laings Status als „Groovy Scientist“ zeigt sich zudem in der großen medialen Aufmerksamkeit, welche ihm zuteilwurde. Dazu gehörten neben Zeitungs- und Magazinartikeln von Underground- sowie Mainstreammedien auch Radio- und Fernsehauftritte (Chapman 2019b). Wie Jakob Tanner (1998: 212) zeigt, kam den Massenmedien eine äußerst wichtige Rolle in der „Multiplikation und Ausbreitung“ gegenkultureller Aktivität zu. Das mediale Interesse an Laing gab ihm die Möglichkeit, sich mit wissenschaftlichen Positionen, die an gesellschaftspolitische Implikationen gekoppelt waren, an ein breites Publikum zu richten. Insofern wurde Laings Position als Ikone der Gegenkultur auch durch die Massenmedien geschaffen.

Den Höhepunkt seiner Bekanntheit erreichte Laing wohl in den frühen 1970er Jahren, als er in den USA auf Vorlesungstournee durch die Universitätscampus ging und dabei ganze Turnhallen füllte (Chapman 2019a). In diesem Zeitraum erschienen auch zwei Filme, denen eine besondere Bedeutung bei der Verbreitung und Popularisierung von Laings Schizophrenieverständnis zukam: Der Spielfilm family life^30^ von Ken Loach, bei dem Laing beratend zur Seite gestanden hatte und auf dessen Schriften der Film basierte, sowie der Dokumentarfilm asylum von Peter Robinson, in dem eine therapeutische Gemeinschaft der Philadelphia Association zusammen mit Laing portraitiert wurde.^31^ Ferner popularisierten Portraits von Laing in der *New York Times* und den Magazinen *Esquire* und *Life *Anfang der 1970er Jahre Laings Theorien über Schizophrenie. Der Artikel im *Life*-Magazin bestand aus einer halben Seite Text und viereinhalb Seiten Fotostrecke, die etwa Laing beim Kopfstand, Laing im abgedunkelten Arbeitszimmer und den barfüßigen Laing auf einem Baum sitzend zeigte. Seine Ideen, so scheint es, waren bereits bekannt, im Vordergrund stand nunmehr seine Person. Laing war zu einem Star der Gegenkultur geworden, der als Kuriosum in den Mainstream-Medien seinen Platz fand. Trotzdem durfte auf dieser halben Seite Laings Kernthese nicht fehlen: Die Grenze zwischen Wahnsinn und psychischer Gesundheit könne in einer Gesellschaft, die in den letzten 50 Jahren rund 100 Millionen ihrer Mitmenschen getötet habe, nicht klar gezogen werden; Schizophrenie könne statt eines „breakdowns“ auch als „breakthrough“ verstanden werden (Haynes 1971: 87). Trotz Laings Abkehr von der konventionellen Psychiatrie fanden also seine Texte und Theorien, die auf seine Forschung Anfang der 1960er Jahre zurückgehen, weiterhin Verbreitung und erreichten gar ein weit größeres Publikum. Ironischerweise hatte Laing – zumindest vordergründig – zu diesem Zeitpunkt nach einem längeren Aufenthalt in Sri Lanka, Indien und Japan, wo er unter anderem Yoga und Meditation gelernt hatte, sowohl das Interesse an der Schizophrenie als auch an der Politik (oder jeglicher Form schärferer Gesellschaftskritik) verloren (Wall 2017: 16; Mezan 1972).

## Fazit

Laing wurde im Verlauf der 1960er Jahre zunehmend gesellschaftskritisch, er entfernte sich aus dem akademischen Umfeld und begann sich politisch zu engagieren. Er wurde, bedingt einerseits durch seine persönliche Entwicklung und Erfahrung, sein gesellschaftliches Umfeld und andererseits durch institutionelle Zwänge und mediale Logiken, vom Psychiater zum Anti-Psychiater. Laing stellte die Gesellschaft und die Rolle der Psychiatrie radikal in Frage, indem er erklärte, als „schizophren“ Diagnostizierte seien näher an ihrem „wahren Selbst“ als die von sich entfremdete Gesellschaft, die sie als „krank“ etikettiert hatte. Basierend auf dieser Überzeugung engagierte er sich für die „Befreiung“ des kollektiven Bewusstseins, etwa durch die Organisation der „Dialectics of Liberation“-Tagung und für einen anderen Umgang mit Menschen, die als psychisch krank erachtet wurden, zum Beispiel durch die Gründung der therapeutischen Gemeinschaft Kingsley Hall. Seine Kritik teilte er ab Mitte der Sechzigerjahre vermehrt außerhalb von psychiatrischen Fachkreisen mit einer breiteren, links-intellektuellen Öffentlichkeit.

Sein Schizophrenie-Verständnis und seine Gesellschaftskritik entwickelte Laing jedoch nicht abseits der Psychiatrie. Sie entstanden, wie ich anhand seiner Schizophrenie-Forschung mit Esterson gezeigt habe, in einem wissenschaftlichen Rahmen. Es handelte sich dabei gleichsam um eine politische Zuspitzung von Forschungsresultaten, die er ähnlich bereits Mitte der 1950er Jahre formuliert hatte. Laings politische Radikalisierung und sein gradueller Rückzug aus der akademischen Wissenschaft hatte mit mehreren Faktoren zu tun. Erstens verlor er zugunsten seines politischen Aktivismus das Interesse an der wissenschaftlichen Forschung. Es gibt zweitens auch Hinweise dafür, dass seine Glaubwürdigkeit aufgrund seines Engagements beim traditionelleren Teil der „scientific community“ litt. Drittens wurde sein Denken in den 1960ern stark von explizit politischen und gesellschaftskritischen Philosophen wie Sartre und Marcuse beeinflusst. Diese Einflüsse und die Themen, die Laing in seinen Texten zu Schizophrenie verhandelte, wie die Repression durch eine konformistische Gesellschaft und die Suche nach einer transzendentalen Wahrheit in der radikalen Subjektivität, erwiesen sich viertens als enorm anschlussfähig an die Gegenkultur der 1960er Jahre und ermöglichten es ihm, eine wirkmächtige Position als „Groovy Scientist“ mit einer psychiatriekritischen gesellschaftspolitischen Positionierung einzunehmen. Schließlich konnte sich Laing fünftens durch die Nutzung neuer Kommunikationskanäle und das Interesse der Massenmedien an seiner Person Gehör verschaffen und fand so ein größeres Publikum für seine Botschaft(en).

## Footnotes

1 Flyer „Dialectics of Liberation“, in: *Dialectics Introduction: Dialectics of Liberation. A Unique Gathering to Demystify Human Violence in all its Forms,* http://www.dialecticsofliberation.com/1967-dialectics/dialectics-introduction/ (01.12.2020).

2 Die Gruppe bestand nur aus Männern.

3 Die Tavistock Research Unit war vom Psychiater und Begründer der Attachement Theory, John Bowlby gegründet worden und stand zu diesem Zeitpunkt noch unter seiner Leitung.

4 Ich folge dem Historiker Oisín Wall (2017: 21) in seiner Verwendung der Bezeichnungen „Anti-Psychiatrie“ und „Anti-Psychiater“, um die Psychiatriekritikwelle der 1960er und 1970er Jahre und deren Akteure zu beschreiben. Die Anti-Psychiatrie-Bewegung war sehr heterogen und nur lose vernetzt. Besonders einflussreich war sie in Großbritannien, Italien, Frankreich und in den USA, es gab aber auch Vertreter*innen in anderen Ländern. Wichtige anti-psychiatrische Positionen waren, dass Wahnsinn sozial konstruiert und sozial bedingt sei; dass psychiatrische Anstalten pathogen seien; und dass über die Psychiatrie Kontrolle ausgeübt werde (Majerus 2010). Im Folgenden geht es in erster Linie um die britische Anti-Psychiatrie-Szene in London, zu der auch nicht-britische Psychiater gehörten und die auch in den USA stark rezipiert wurde.

5 Peter Fischer: Die kranke Seele. Das geteilte Selbst in der Praxis des Dr. Laing. Ein Bericht aus London. Sendung des Hessischen Rundfunks vom 17.11.1976.

6 Gegenüber Paul Mezan (1972) sagte er: „Politically, I think I’m neutral really. I engage in no strictly *political* actions—except in the sense of following the Tao.“

7 Vergleiche zu soziogenen Erklärungen in der Psychiatrie nach 1945 insbesondere Staub (2011) und Harrington (2019). Zur Hypothese, dass Familien Schizophrenie verursachen vgl. Hartwell (1996), Weinstein (2013) und Lienhard (2020).

8 Zu einem Verkaufsschlager wurde es aber erst, als 1965 eine Taschenbuchauflage bei Penguin erschien.

9 Staub (2011: 46) meint: „In breadth and range, the effect this slim essay by Bateson’s team was to have proved almost incalculable.“ Ebenfalls prägend für diese Forschungsrichtung waren etwa Publikationen von den Teams um Theodore Lidz, Lyman Wynne und Harold Searles.

10 Bateson et al. vertraten im Aufsatz (1956) die These, dass Schizophrene auf verschiedenen Kommunikationsebenen (z. B. verbal/nonverbal) widersprüchliche Botschaften ihrer Eltern bekämen. Da diese Form von Kommunikation keine richtigen Antworten ermögliche, führe eine solche „doppelte Bindung“ zur Ausprägung schizophrener Symptome. Während sie 1956 noch zwischen „bindenden“ und „gebundenen“ Personen unterschieden, beschrieben sie in späteren Jahren „schizophrene Familien“ als selbsterhaltendes System ohne organisierendes Zentrum.

11 Vgl. zum geplanten Projekt: Laing, Ronald D. *Draft Application for a Grant for The Study of Schizophrenic Patients and Their Family Interactions, *01.1960, University of Glasgow Archives and Special Collections, GB 247, MS Laing, L436/2, S. 6.

12 Esterson, Aaron und Ronald D. Laing. *Family and Schizophrenia: Research and Therapy. *Application for Funds by Drs. A. Esterson and R. D. Laing, ca. 1963, University of Glasgow Archives and Special Collections, GB 247, MS Laing, A627, hier: S. 1.

13 Laing, Ronald D. *Application to the Foundations Fund for Research in Psychiatry: Towards a Phenomenology of Family Life,* 03.1964, University of Glasgow Archives and Special Collections, GB 247, MS Laing, A627, hier: S. 8.

14 Noch im Oktober 1965, ein Jahr nach dem Erscheinen des ersten Bandes, schrieb Laing in einem Jahresbericht für das Tavistock Institute, die Familienforschung mit normalen und schizophrenen Familien gehe weiter und laufe gut, *Sanity, Madness and the Family. Volume II* sei in Planung. Vgl. Laing, Ronald D. *Draft Annual Report of the Tavistock Institute of Human Relations.* 30 Sep. 1964–30 Sep. 1965. Schizophrenia and Family Research Unit., 10.1965, University of Glasgow Archives and Special Collections, GB 247 MS Laing V224.

15 Im Vorwort zur Neuauflage 1969 erklärten Laing und Esterson, sie hätten sich bewusst gegen die Verwendung einer Kontrollgruppe entschieden, da eine solche nichts mehr zu ihrer Forschungsfrage hätte beitragen können. Vgl. Laing, Ronald D. und Aaron Esterson. *Sanity, Madness and the Family: Families of Schizophrenics *[1964], Harmonsdsworth 1972, S. 13.

16 Laings Frau hatte sich von ihm getrennt und er arbeitete sehr intensiv in seiner Privatpraxis (Laing & Mullan 1995: 284, 177).

17 Laing Ronald D. *Draft Annual Report of the Tavistock Institute of Human Relations.* 30 Sep. 1964–30 Sep. 1965. Schizophrenia and Family Research Unit., 1965.

18 Speck, in: *Conference on „Society and Psychosis“*. 14.–15. Oktober 1966, Special Collections Glasgow, MS Laing A, MS Laing A627, hier: S. 3–4.

19 Laing, Ronald D., in: *Conference on „Society and Psychosis“*, 15.10.1966, University of Glasgow Archives and Special Collections, GB 247 MS Laing A627, hier: S. 131–132.

20 Die Neuauflage 1969 erschien bei Penguin und dürfte dadurch ein größeres Publikum erreicht haben. Zur Rolle von Penguin in der Verbreitung von sozialkritischen Psychiatrietheorien vgl. Miller (2015).

21 Laing, Ronald D. *Application to the Foundations Fund for Research in Psychiatry: Towards a Phenomenology of Family Life,* 1964, S. 6.

22 Vgl. z. B. Page, Ian 1964. Review of „Sanity, Madness and the Family“. *Social Work 21* (4): 22–23; Lomas, P. 1965. Review of „Sanity, Madness and the Family: Vol. 1. Families of Schizophrenics“. By R. D. Laing and A. Esterson. *International Journal of Psychoanalysis* 46: 390–392; Wing, John K. 1964. Sanity, Madness, and the Family: Volume 1 – Families of Schizophrenics (Book Review). *New Society* 3 (84): 28–29; Little, A. 1965. Sanity, Madness and the Family (Book Review). *British Journal of Sociology* 16 (3): 277; Kafka, John S. 1966. Intelligible Madness. *Contemporary Psychology: A Journal of Reviews* 11 (10): 472–473.

23 Auch Estersons Karriere änderte sich in der Folge der Publikation des Buchs. Er verließ die Klinik zugunsten einer Privatpraxis (laut eigenen Angaben nicht ganz freiwillig) und beschäftigte sich weiterhin mit Familien Schizophrener. 1972 veröffentlichte er eine vertiefende Studie einer der in *Sanity, Madness and the Family *porträtierten Familien: *The Leaves of Spring* (McGeachan 2014: 290–291).

24 Das Vorwort ist mit September 1964 datiert, erschien aber erst 1965.

25 Cooper hatte bereits 1962 die „Villa 21“ gegründet, eine experimentelle Abteilung des Shenley Hospital in Hertfordshire, die er als eine Art therapeutische Gemeinschaft betrieb. Die Villa 21 wurde als „anti-hospital“ bekannt und wurde von Cooper (1967) in seinem Buch *Psychiatry and Anti-psychiatry* beschrieben. Vgl. dazu auch Wall (2013).

26 Laing bezog sich hier direkt auf Freuds *Unbehagen in der Kultur*, seine Lektüre war jedoch offensichtlich von der jüngeren linken Rezeption geprägt.

27 Der Artikel ist ein Abdruck eines Referats, das Laing an der ersten internationalen Tagung für Sozialpsychiatrie hielt (siehe Hinweis in der Publikation).

28 Die Ursprünge des New Age lassen sich bis ins 18. Jahrhundert zurückverfolgen. Steven Sutcliffe (2003) führt die Anfänge des „neuen“ New Age in Großbritannien auf die 1930er zurück.

29 Flugblatt „Dialectics of Liberation“, zitiert nach: Dialectics Introduction, *Dialectics of Liberation. A Unique Gathering to Demystify Human Violence in all its Forms*, http://www.dialecticsofliberation.com/1967-dialectics/dialectics-introduction/ (01.12.2020).

30 family life basierte auf dem Fernsehfilm in two minds, den Loach zusammen mit dem Drehbuchautor David Mercer und dem Produzenten Tony Garnett 1967 gedreht hatte. Der Fernsehfilm wurde sehr kritisch aufgenommen und David Mercer bestritt damals in der BBC-Talkshow „Late Night Up“, sich von Laings Ideen inspiriert haben zu lassen. Sechs Jahre später, als wohl der soziale Druck gewichen war, gab Mercer gegenüber *Theatre Quarterly* zu, dass Laing der offizielle Berater für den Film gewesen sei. Vgl. El Mubarak Mustafa (1981) und Moat (o.J.). Wie aus einem Briefwechsel zwischen dem Produzenten Irving Teitelbaum mit Laings Sekretärin Mary Simon hervorgeht, war Laing auch für family life beratend tätig, außerdem hätten seine Schriften als Vorlage für den Film gedient. Vgl. Teitelbaum, Irving. *Schreiben an Mary Simon,* o. D., University of Glasgow Archives and Special Collections, GB 247 MS Laing L238/121–124.

31 Ken Loach: *Family Life, *Drama, 1 h48 m, EMI Films, Kestrel Films, 18.02.1972; Peter Robinson: *Asylum,* Documentary, 1h35 m, Peter Robinson Associates, Surveillance Films, 12.03.1975.
